# Lack of Epileptogenic Effects of the Creatine Precursor Guanidinoacetic Acid on Neuronal Cultures In Vitro

**DOI:** 10.3390/biom13010074

**Published:** 2022-12-30

**Authors:** Fabio Poggio, Martina Brofiga, Mariateresa Tedesco, Paolo Massobrio, Enrico Adriano, Maurizio Balestrino

**Affiliations:** 1 Department of Informatics, Bioengineering, Robotics, and Systems Engineering (DIBRIS), University of Genova, 16145 Genova, Italy; 2 3Brain Gmbh, 8820 Wadenswil, Switzerland; 3 National Institute for Nuclear Physics (INFN), 16146 Genova, Italy; 4 Department of Neuroscience, Rehabilitation, Ophthalmology, Genetics and Maternal and Child Sciences (DINOGMI), University of Genoa, 16145 Genova, Italy; 5 IRCCS Ospedale Policlinico San Martino, 16145 Genova, Italy

**Keywords:** guanidinoacetic acid, creatine, GAMT deficiency, seizures, epilepsy, electrophysiology

## Abstract

The creatine precursor Guanidinoacetic Acid (GAA) accumulates in the genetic deficiency of the GuanidinoAcetate Methyl Transferase (GAMT) enzyme and it is believed to cause the seizures that often occur in this condition. However, evidence that it is indeed epileptogenic is scarce and we previously found that it does not cause neuronal hyperexcitation in in vitro brain slices. Here, we used Micro-Electrode Arrays (MEAs) to further investigate the electrophysiological effects of its acute and chronic administration in the networks of cultured neurons, either neocortical or hippocampal. We found that: (1) GAA at the 1 µM concentration, comparable to its concentration in normal cerebrospinal fluid, does not modify any of the parameters we investigated in either neuronal type; (2) at the 10 µM concentration, very similar to that found in the GAMT deficiency, it did not affect any of the parameters we tested except the bursting rate of neocortical networks and the burst duration of hippocampal networks, both of which were decreased, a change pointing in a direction opposite to epileptogenesis; (3) at the very high and unphysiological 100 µM concentration, it caused a decrease in all parameters, a change that again goes in the direction opposite to epileptogenesis. Our results confirm that GAA is not epileptogenic.

## 1. Introduction

**Figure 1:**
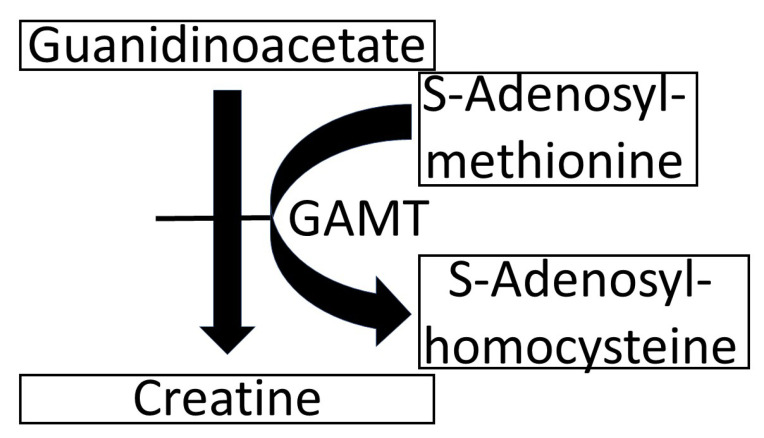
Synthesis of creatine from guanidinoacetate, catalyzed by the enzyme GuanidinoAcetate Methyl Transferase (GAMT).

Guanidinoacetic Acid (GAA) is the precursor of creatine, which it forms through the reaction depicted in Figure 1 [1]. In the rare genetic disease, it is characterized by the deficiency of the enzyme that catalyzes the conversion of GAA to creatine (GuanidinoAcetate Methyl Transferase—GAMT—see Figure 1). In GAMT deficiency, GAA in the cerebrospinal fluid accumulates and reaches 11–13 µM, compared with the normal range of 0.068–0.114 µM. [2]. It has been suggested that this GAA increase is responsible for the seizures that often occur in GAMT deficiency [3]. At first glance, this causal relationship may be challenged by the fact that seizures are absent from the clinical picture in 19% of cases, despite the constant increase in GAA [2,4]. Finally, we recently demonstrated that GAA does not cause epileptic-like hyperexcitability when applied acutely to brain slices in vitro [5]; an observation that further challenges the notion that GAA may be epileptogenic. Although GAA may still cause epilepsy through alterations in neuronal sprouting [6], it would be important to understand whether such epileptogenicity in GAMT-deficient patients, if it really exists, may be due exclusively to long-term effects in the developing brain or also to a direct neuronal excitation by GAA. In the present paper, we follow up our previous findings [5] by further investigating whether GAA may cause hyperexcitability in neuronal networks cultured in vitro. To achieve this goal, we designed experimental protocols, where cortical and hippocampal neuronal networks were coupled to Micro-Electrode Arrays (MEAs) to investigate the electrophysiological effects of GAA acute and chronic administration by modulating the emerging spiking and bursting activity.

## 2. Materials and Methods

### 2.1. Cell Culture Preparation

Dissociated cortical and hippocampal cultures were prepared as follows: Cortices and hippocampi of (E18) rat embryos were finely chopped. The tissues were digested with 0.125% trypsin (Sigma Aldrich, St. Louis, MO, USA) and 0.05% DNAse (Sigma Aldrich) in Hank’s Balance Salt Solution (Sigma Aldrich) without calcium and magnesium for 18 min at 37 °C. The digestion was halted by adding fetal bovine serum (FBS, 10%, Sigma Aldrich, St. Louis, MO, USA) complemented medium (Neurobasal). This step was followed by mechanical dissociation by trituration. Cells were resuspended in Neurobasal medium (Gibco Invitrogen, Waltham, MA, USA), supplemented with 2% B-27 supplement (Gibco Invitrogen), 1% stable L-Glutamine (GlutaMAX 100× Gibco Invitrogen), 1% PenStrep (Penicillin–Streptomicin Solution, Gibco Invitrogen), and plated on the already poly-L-ornithine- (100 µg/mL, Sigma Aldrich) coated MEAs at the final density of 1′500 cells/mm^2^ and 1′300 cells /mm^2^ for the cortical and hippocampal cultures, respectively. The cultures were maintained at 37 °C with 5% CO_2_ and 95% humidity. After 5 days, and twice a week afterward, a half volume of the medium was replaced with BrainPhys medium, supplemented with 2% NeuroCult SM1, 1% Glutamax, and 1% PenStrep solution.

### 2.2. Ethical Approval

The experimental protocol for in vitro cultures was approved by the European Animal Care Legislation (2010/63/EU) and the Italian Ministry of Health, in accordance with D.L. 116/1992 and the guidelines of the University of Genova (Prot. 75F11.N.6JI, 08/08/18).

### 2.3. Experimental Protocols

To test the effect of GAA, we tested three different experimental conditions and, for each of them, we recorded the electrophysiological activity of the neuronal cultures outside the incubator, using the MEA2100 system (Multi Channel Systems, MCS, Reutlingen, Germany) with a sampling frequency of 10 kHz. The Micro-Electrode Arrays (MEAs) employed in the present work consisted of 60 flat round electrodes (MCS), with a diameter of 30 µm arranged in an 8 × 8 square grid, with the four corners missing and a distance of 200 µm between adjacent electrodes. Environmental factors conditioning the neuronal networks were kept constant over the recording: the temperature was set at 37 °C, while the flow of gas was kept at 5% CO_2_, 20% O_2_, and 75% N_2_. MC_Rack software (Multi Channel Systems, MCS, Reutlingen, Germany, version 4.6.2) was exploited both to record data and to deliver electrical pulses to individual electrodes.

Acute GAA stimulation: After 18 Days In Vitro (DIV), we delivered GAA to the culture medium at increasing concentrations (1 µM, 10 µM, and 100 µM). For each concentration, the electrophysiological activity was recorded for 10 min. For this experimental condition, we performed *n* = 8 recordings for cortical and *n* = 7 recordings for hippocampal neurons. For each neuronal type, we also recorded control experiments (i.e., GAA not delivered to the culture). The cultures used for this protocol came from 4 rats.

Chronic GAA stimulation: We delivered 10 µM of GAA on the day of the plating, keeping the same concentration for the entire lifetime of the neuronal networks. We applied this protocol only to the cortical assemblies. For 20 min, we recorded their electrophysiological activity at DIV 11, 18, and 25. We compared these results with those obtained from the cultures without the delivery of GAA (controls). This protocol made use of *n* = 4 cortical networks treated with GAA and *n* = 5 controls (all cultures from the same rat).

Acute GAA delivery combined with electrical stimulation: We electrically stimulated the neuronal networks before and after the administration of GAA at 10 µM, repeating the protocol at DIV 11 and 18. Each recording phase lasted 40 min. First, 15 min of spontaneous activity was acquired to characterize the network dynamic in the basal condition (i.e., in the absence of both chemical and electrical stimuli). Then, we delivered the electrical stimuli for 5 min. Such a stimulation protocol consists of a biphasic electrical stimulus lasting 40 µs with a 50% duty cycle (positive phase first), with a frequency of 0.2 Hz. Then, 10 µM of GAA was delivered and the resulting dynamic was recorded for 15 min. Eventually, the electrical stimulation was then repeated for 5 min.

### 2.4. Data Analysis

#### 2.4.1. Spike Detection

The first step to be carried out in the study of electrophysiological signals is the identification of the spikes. To discriminate the occurrence of spike events, we used the Precise Timing Spike Detection (PTSD) [7]. To work properly, such an algorithm requires specific parameters: the Peak Lifetime Period (PLP, set at 2 ms) and the Refractory Period (RP, set at 1 ms), which refer to the duration of a spike and the minimum interval likely to elapse between two consecutive events, respectively. An additional parameter necessary for this analysis is the differential threshold (DT), which is set independently for each channel and computed according to the standard deviation of the biological and thermal noise of the signal (we set DT as 8 times the standard deviation of noise).

#### 2.4.2. Burst Detection

To extract bursts for all possible arrangements of high-frequency spike train behavior, we used the string method devised in [8]. Bursts defined as nearly vertical strings of spikes require only two parameters: (i) the minimum number of spikes (*N_s_* = 5) that a string must have before it is accepted as a burst; (ii) the maximum inter-spike interval (*t_s_ =* 100 ms) that elapses between two adjacent spikes into a burst.

#### 2.4.3. Spiking and Bursting Analysis

Spike sorting on data was not performed, since it would be limited by the high number of neurons whose activity was recorded from a single electrode. The sum of all the spikes recorded through one only channel and normalized with respect to the temporal window of observation represents the Mean Firing Rate (MFR) of the channel. Once this is computed for each channel, the MFR of an entire neuronal population could be extracted by calculating the average of MFRs related to all channels. The firing rate threshold was set at 0.1 spikes/s.

Once the burst train was identified, we computed the Mean Bursting Rate (MBR), i.e., the number of detected bursts per minute. The bursting rate threshold was set at 0.4 bursts/min. The bursting behavior can be quantitatively characterized also by other parameters such as the Burst Duration (BD), expressed in ms, and the Mean Frequency Intra-Burst (MFIB), i.e., the number of detected spikes into a burst per ms. Bursting parameters for each electrode were averaged to extract the MBR, the BD, and the MFIB of the entire neuronal culture.

#### 2.4.4. Electrical Stimulation Analysis

To quantify the effect of the electrical stimulation on the neuronal activity, we computed the Post-Stimulus Time Histogram (PSTH). To do that, we considered time windows of 600 ms following each stimulus, divided into 4 ms bins. Then, we computed the number of spikes within each time bin. For each electrode, we extracted the area under the PSTH curve and the latency of the peak. Finally, we derived the corresponding quantities for the entire neuronal culture by averaging all values for each electrode. Electrodes whose PSTH area was lower than “1” (i.e., less than one evoked spike) were considered inactive; then, they were removed from the analysis.

## 3. Results

### 3.1. Effects of Various Concentrations of GAA on Electrophysiological Parameters

**Figure 2:**
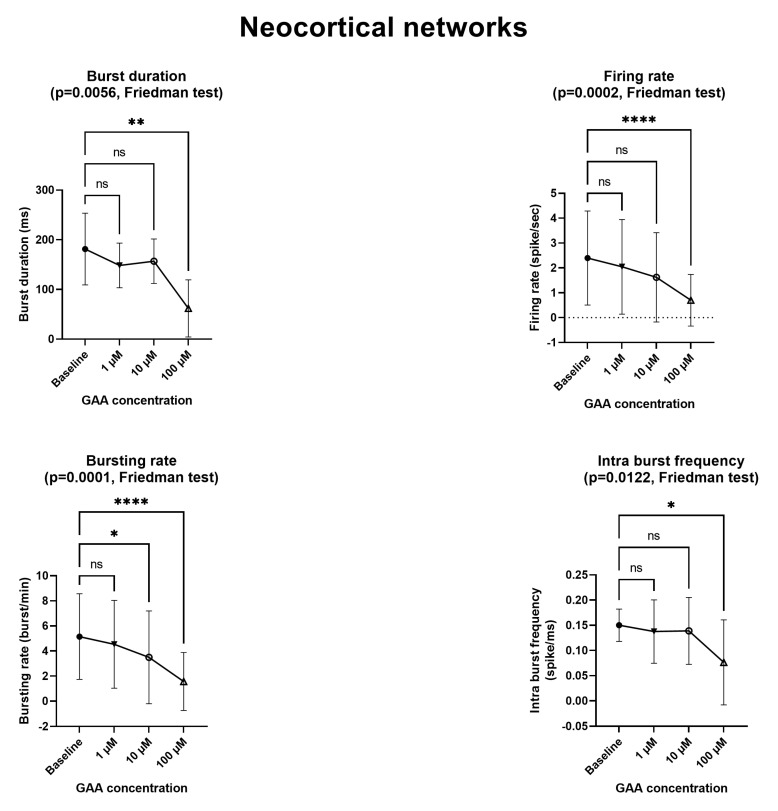
Effects of Guanidinoacetic Acid (GAA) on the spiking and bursting parameters in neocortical networks. All parameters refer to spontaneous—not stimulus-evoked—activity. Symbols and bars represent mean and standard deviation. Asterisks mark statistically significant differences from baseline (number of asterisks represent number of decimal zeroes before non-zero number, e.g., *** = *p* < 0.0005). ns—not significant. See text for additional explanations.

**Figure 3:**
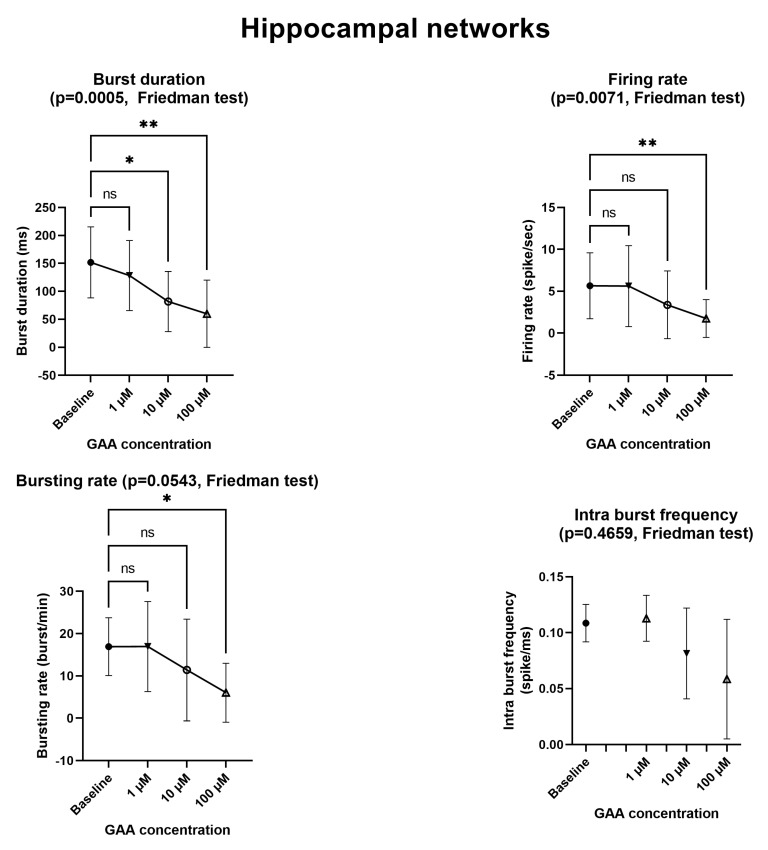
Effects of Guanidinoacetic Acid (GAA) on the spiking and bursting parameters in hippocampal networks. All parameters refer to spontaneous—not stimulus-evoked—activity. Symbols and bars represent mean and standard deviation. Asterisks mark statistically significant differences from baseline (number of asterisks represent number of decimal zeroes before non-zero number, e.g., ** = *p* < 0.005). ns—not significant. See text for additional explanations.

Figure 2 and Figure 3 and Supplemental Tables S1–S4 summarize the effects of various concentrations of GAA on the spiking and bursting parameters of neuronal networks (cf., Sect. 2.5) made either of neocortical neurons (Figure 2) or of hippocampal neurons (Figure 3). At the 1 µM concentration, GAA had no effect on any parameter in either neuronal type. It should be noted that this concentration is close to the 0.068–0.114 µM concentration that is found in the cerebrospinal fluid of normal subjects [2]. At the 10 µM concentration, it significantly decreased the bursting rate of the neocortical neurons and the burst duration of the hippocampal neurons while it still had no significant effect on any other parameter in either type of neurons. It should be noted that this 10 µM concentration is very close to the concentration that occurs in the cerebrospinal fluid of patients affected by GuanidinoAcetate Methyl Transferase (GAMT) deficiency, where an 11–12 µM concentration of GAA is found [2]. At the very unphysiological concentration of 100 µM, GAA decreased all parameters in both neuron types, except the intra-burst frequency in the hippocampal networks.

### 3.2. Effects of 10 µM GAA Concentration on Electrophysiological Parameters at Various Culture Ages

We asked whether the effects of GAA might be different as a function of the neuronal culture development. Given the substantial similarity of the results we had obtained in the neocortical and hippocampal networks (see above, Figure 2 and Figure 3), we carried out these experiments only in one neuronal type, namely the neocortical networks.

**Figure 4:**
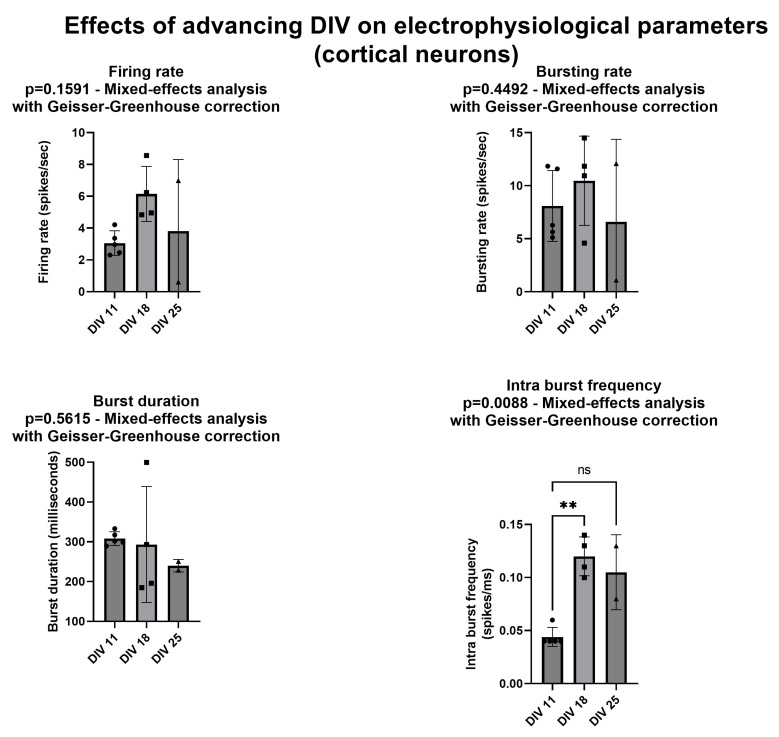
Effects of DIV on the spiking and bursting parameters. All parameters refer to spontaneous—not stimulus-evoked—activity. Bars and lines represent mean and standard deviation. Filled circles represent individual data. Asterisks mark statistically significant differences from DIV 11 (number of asterisks represent number of decimal zeroes before non-zero number, e.g., ** = *p* < 0.005). ns—not significant. See text for additional explanations.

First, we investigated whether the selected electrophysiological parameters were affected by the days in culture (or DIV) of the neuronal network. To this aim, we prepared five neocortical networks and tested them at DIV = 11, DIV = 18, and DIV = 25. We chose these DIVs to document possible changes in network development based on our previous studies on cortical and hippocampal cultures coupled with MEAs [9]. It should be noted that, while all cultures were viable at DIV 11, one of them was no longer viable at DIV 18 and two additional ones were no longer viable at DIV 25. Thus, we could not use regular ANOVA for paired data, but instead we used repeated measures ANOVA mixed-effect analysis with a Geisser–Greenhouse correction, as carried out by the Prism software we used (GraphPad Prism version 9.4.1.681 for Windows, GraphPad Software, San Diego, CA, USA, www.graphpad.com, released on 21 July 2022). Figure 4 and Supplemental Table S5 show that the firing rate, the bursting rate, and the burst duration were not affected by the DIV of the culture, while the intra-burst frequency significantly increased between DIV 11 and DIV 18; it remained elevated to the same extent at DIV 25, even if the difference was no longer significant compared with the baseline.

**Figure 5:**
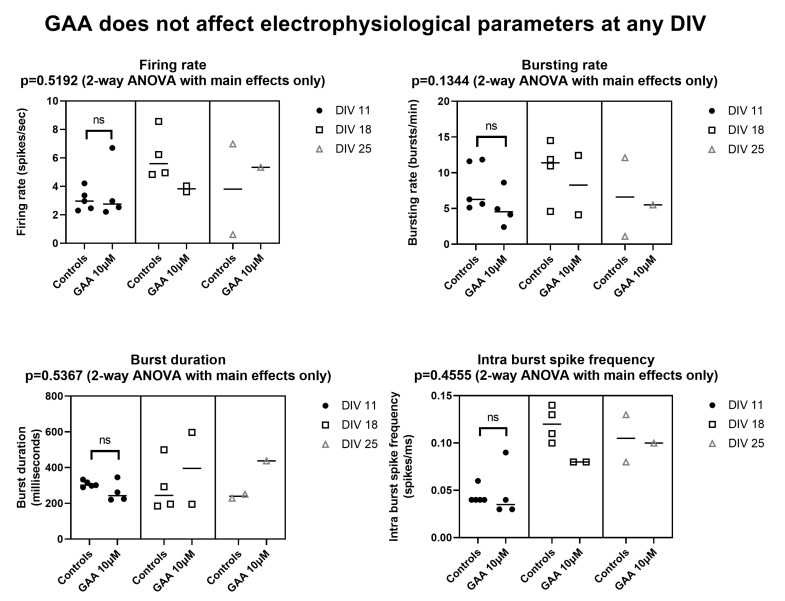
Effects of 10 µM Guanidinoacetic Acid (GAA) on the spiking and bursting parameters in neocortical networks. All parameters refer to spontaneous—not stimulus-evoked—activity. Symbols represent individual experiments. Horizontal bars represent medians. ns—not significant (Mann–Whitney test). See text for additional explanations.

Next, we asked whether GAA at the 10 µM concentration (which is very close to the concentration of this metabolite that is found in the cerebrospinal fluid of GAMT-deficient patients [2]) had any effect on the various parameters at the various ages of the cultures. To answer this question, we delivered GAA at a 10 µM concentration during the day of the preparation. Figure 5 and Supplemental Table S5 show that we found no such effect on any parameter at any of the culture ages we tested. It should be noted that at DIV 11 we compared five control networks with four GAA-treated ones. Due to decreased network viability, at DIV 18 we compared four and two cultures, respectively, and at DIV 25 we compared two and one networks. Thus, for statistical analysis we used Analysis of Variance (ANOVA) for unpaired data with main effects only, as carried out by the Prism software we used (GraphPad Prism version 9.4.1.681 for Windows, GraphPad Software, San Diego, California USA, www.graphpad.com, released on 21 July 2022). Unfortunately, the above-mentioned limited culture viability left us with very small samples for DIV 18 and for DIV 25 (for DIV 18, four controls and two treated; for DIV 25, two controls and one treated, see Figure 5). While we acknowledge that this is a limitation of our results, we decided to show these data anyway, not only because a statistic is anyway possible (see Figure 5), but especially because, even with the obvious limitation of the very small sample size, they do not suggest that GAA is epileptogenic. In other words, the albeit scarce number of observations does not suggest greater hyperexcitability than the controls. The data for DIV 11 are, moreover, sufficient to further demonstrate the lack of hyperexcitability by GAA. In fact, at this culture age, no significant differences were found by the Mann–Whitney test between the controls and the treated cultures (see Figure 5).

### 3.3. Effects of 10 µM GAA on Stimulus-Evoked Spikes

**Figure 6:**
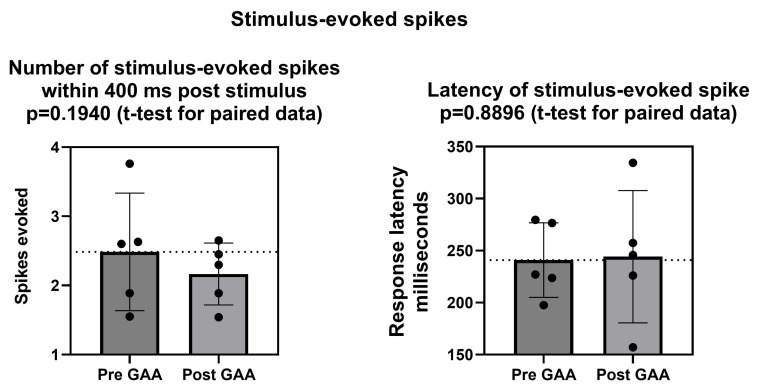
Effects of 10 µM Guanidinoacetic Acid (GAA) on number and latency of evoked spikes in neocortical networks. Bars and lines represent mean and standard deviation. Symbols represent individual experiments. See text for additional explanations.

Finally, we asked whether GAA had any effect on the response of cortical networks to electrical stimulation. We chose cortical networks because, in previous work, we found that they displayed a better responsiveness than hippocampal ones [10]. Figure 6 shows that neither the number of stimulus-evoked spikes nor the latency of the stimulus-evoked responses were affected by addition to the culture medium of 10 µM GAA.

## 4. Discussion

Epileptogenic agents modify, in a hyperresponsive direction, the excitability of neuronal networks of cultured neurons in vitro. For example, using Micro-Electrode Arrays (MEAs) similar to those used in this study, Sokal et al. [11] found that gabazine (an antagonist of GABAA receptors) increases the mean firing rate. McSweeney et al. [12] described an in vitro model of epilepsy in which (among others) the mean firing rate and the bursting rate where increased, again using MEAs similar to ours. By contrast, we found that GAA—a supposed epileptogenic agent—does not modify any of the parameters we tested (Figure 2 and Figure 3) at the concentration of 1 µM, which is comparable to that of normal cerebrospinal fluid [2]. At the concentration of 10 µM, which is very close to the 11–13 µM concentration that is found in GAMT-deficient patients [2], it still did not change any parameters, with the exceptions of the bursting rate of the neocortical networks (Figure 2) and the burst duration of the hippocampal networks (Figure 3), both of which were decreased (a change that goes in a direction opposite to that of epileptogenesis). In fact, the two changes we found at the 10 µM concentration indicate decreased rather than increased excitability and spontaneous firing. Furthermore, at the 100 µM concentration, GAA decreased all electrophysiological parameters (Figure 2 and Figure 3), again pointing in an opposite direction to what would be expected from an epileptogenic agent.

These results confirm our previous findings [5], showing that GAA does not change the amplitude of the postsynaptic compound action potential (“population spike”) in mouse hippocampal slices, a model where epileptogenic agents do cause a large increase in such amplitude [13,14]. The fact that GAA does not change electrophysiological parameters of in vitro neural circuits like known epileptogenic agents do argues strongly against the idea that it is an epileptogenic agent, at least when acutely applied to neural tissue.

The fact that GAA does not act as an epileptogenic agent may explain why seizures are not constant in all GAMT-deficient patients, despite the consistency of the GAA increase in all of them [2,4].

However, the possibility remains that GAA may cause epilepsy in GAMT-deficient patients by causing alterations in neuronal sprouting, as described by Hanna-El-Daher et al. [6]. Confirming this hypothesis would require a longer-lasting administration of GAA, compatible with the development of the altered network wiring that GAA causes [6].

**Figure 7:**
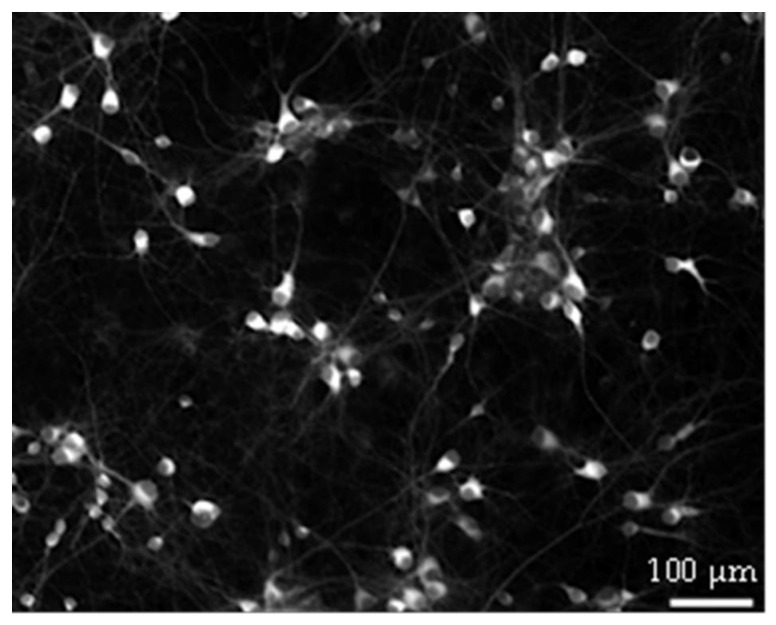
Example of hippocampal neurons plated on Micro-Electrode Array (MEA) at DIV 18. Neurons established a dense connectivity which made it hard to solve their morphology.

Further research is needed to explain why GAA causes a decrease in the above-mentioned parameters upon its acute addition at the 10 µM concentration and of all the parameters at the 100 µ concentration. One possibility is that GAA at these concentrations may have some hypothetical still unknown toxic effect on neurons. However, it is hard to provide an answer to this question with the current configuration. To generate a sustained spontaneous activity, neurons must be plated over the MEA surface at high density (about 1200–1500 cells/mm^2^, see Figure 7). At this density, it is impossible to solve the neuronal morphology by using both the differential interference contrast microscopy and the immunofluorescence technique; thus, it becomes impossible to appreciate possible variations/alterations in the morphology at the single neuron level.

Our present results, together with those we recently published [5], strongly suggest that if GAA really causes epilepsy in GAMT-deficient patients it does not do so by increasing the excitability of neuronal networks, even if the possibility remains that it may cause epilepsy in such patients by altering the development of neuronal connections, as Hanna-El-Daher et al. have shown [6]. This modifies our knowledge of the effects of GAA on the human brain by suggesting that epileptogenesis by GAA (if really confirmed) is a long-term effect, not an effect that takes place or may be modified in the short term. In fact, modifications in neurite sprouting and the consequent network wiring require at least many days to take place and, even if they were reversible, they would not be so in the short term. Moreover, this may explain why the administration of GAA is well-tolerated by adult subjects, in whom changes in neuronal wiring are probably not so continuous as those that take place in developing brains, as reported by some clinical trials that were recently reviewed by Ostojic [15].

Finally, the hypothetical possibility exists that, in theory, the brain of GAMT-deficient patients may be—due to the lack of creatine—more susceptible to effects of GAA that are not apparent in normal brains, as were those of our rats. To the best of our knowledge, no data exist that even suggest such a different susceptibility of creatine-deficient brains; however, this may be an interesting subject for further research.

## 5. Conclusions

Guanidinoacetic Acid (GAA) does not cause seizure-like neuronal hyperexcitation, at least not in the short term, upon acute administration like typical epileptogenic agents do. However, it may still cause epilepsy in the long term by determining altered neuronal sprouting [6]. Important consequences of these notions are that: (1) the decrease in GAA in GAMT-deficient patients would not cause a reduction of seizure frequency in the short term and (2) the adult brain should probably not be susceptible to brain-adverse effects of GAA.

## Data Availability

Data supporting reported results can be found as supplementary tables, as quoted in the text. In addition, the peak trains of the entire dataset of this paper as well as the customized Matlab functions used to analyze the data have been deposited in Zenodo. The DOI of the deposited data and code reported in this paper is https://doi.org/10.5281/zenodo.7429271.

## Supplementary Materials

The following supporting information can be downloaded at: https://www.mdpi.com/article/10.3390/biom13010074/s1, Table S1: spontaneous mean firing rate in neocortical networks. Table S2: spontaneous mean bursting rate in neocortical networks. Table S3: spontaneous mean burst duration in neocortical networks. Table S4: spontaneous mean frequency intra burst in neocortical networks. Table S5: effects of DIV and GAA on various electrophysiological parameters. Table S6: effects of 10 µM GAA on number and latency of stimulus-evoked spikes.
