# Supplementary material for: Lack of Epileptogenic Effects of the Creatine Precursor Guanidinoacetic Acid on Neuronal Cultures In Vitro
Source: Biomolecules. 2022 Dec 30;13(1):74. doi: 10.3390/biom13010074 (PMC9856136; doi:10.3390/biom13010074)
Supplement: Supplementary file 1 [file biomolecules-13-00074-s001.zip › Supplemental table 1.pdf]

SUPPLEMENTAL TABLE 1: Mean firing rate (MFR) of single networks - Measurement unit: spikes/sec

NEOCORTICAL NETWORKS

Internal code number of network	Concentration of guanidinoacetic acid (GAA)			
	Baseline	1 µM	10 µM	100 µM
15345	2,53	1,5	1,6	0,18
18331	5,99	6,05	5,5	2,9
18332	3,46	3	2,95	1,66
19216	1,1	1,24	0,69	0,48
20551	0,64	0,55	0,48	0,11
20559	3,67	2,89	1,05	0,17
22643	0,78	0,33	0,42	0,11
20554	1	0,8	0,29	0

HIPPOCAMPAL NETWORKS

Internal code number of network	Concentration of guanidinoacetic acid (GAA)			
	Baseline	1 µM	10 µM	100 µM
24250	1,52	1,08	0,06	0
20554	2,9	2,52	1,48	0,2
20512	12,32	14,86	9,58	2,38
18333	4,88	6,03	0,81	2,04
18332	2,12	2,06	2,16	1,27
18331	7,85	8,59	8,84	6,36
15345	8,04	4,18	0,73	0
