# Supplementary material for: Lack of Epileptogenic Effects of the Creatine Precursor Guanidinoacetic Acid on Neuronal Cultures In Vitro
Source: Biomolecules. 2022 Dec 30;13(1):74. doi: 10.3390/biom13010074 (PMC9856136; doi:10.3390/biom13010074)
Supplement: Supplementary file 1 [file biomolecules-13-00074-s001.zip › Supplemental table 2.pdf]

SUPPLEMENTAL TABLE 2: Mean bursting rate (MBR) of single networks - Measurement unit: bursts/min

NEOCORTICAL NETWORKS

| Internal code number of network | Concentration of guanidinoacetic acid (GAA) | | | |
|---|---|---|---|---|
| | Baseline | 1 µM | 10 µM | 100 µM |
| 15345 | 4,74 | 3,44 | 2,97 | 0,57 |
| 18331 | 11,4 | 11,77 | 11,23 | 6,39 |
| 18332 | 7,54 | 6,59 | 6,85 | 3,8 |
| 19216 | 3,46 | 4,41 | 2,09 | 1,2 |
| 20551 | 2,06 | 1,86 | 1,73 | 0,4 |
| 20559 | 7,66 | 5,44 | 1,81 | 0,19 |
| 22643 | 1,83 | 1,03 | 0,55 | 0 |
| 20554 | 2,49 | 1,74 | 0,71 | 0 |

HIPPOCAMPAL NETWORKS

| Internal code number of network | Concentration of guanidinoacetic acid (GAA) | | | |
|---|---|---|---|---|
| | Baseline | 1 µM | 10 µM | 100 µM |
| 24250 | 8,05 | 5,89 | 0,29 | 0 |
| 20554 | 16,31 | 12,65 | 8,34 | 0,83 |
| 20512 | 26,11 | 38,88 | 31,96 | 10,25 |
| 18333 | 15,68 | 17,91 | 3,06 | 6,36 |
| 18332 | 10,01 | 9,76 | 9,88 | 5,67 |
| 18331 | 17,18 | 18,36 | 23,95 | 19,16 |
| 15345 | 25,15 | 15,1 | 2,4 | 0 |
