# Supplementary material for: Lack of Epileptogenic Effects of the Creatine Precursor Guanidinoacetic Acid on Neuronal Cultures In Vitro
Source: Biomolecules. 2022 Dec 30;13(1):74. doi: 10.3390/biom13010074 (PMC9856136; doi:10.3390/biom13010074)
Supplement: Supplementary file 1 [file biomolecules-13-00074-s001.zip › Supplemental table 3.pdf]

SUPPLEMENTAL TABLE 2: Mean burst duration (BD) of single networks - Measurement unit: mSec

NEOCORTICAL NETWORKS

Internal code number of network	Concentration of guanidinoacetic acid (GAA)			
	Baseline	1 µM	10 µM	100 µM
15345	193,64	147,59	177	49,55
18331	150,61	143,67	131,64	114,52
18332	153,65	151,56	132,01	128,32
19216	142,31	126,35	133,4	135,85
20551	110,15	99,94	93,1	55,38
20559	202,01	220,43	228,57	11
22643	345,33	93,39	151,46	0
20554	153,72	203,74	207,85	0

HIPPOCAMPAL NETWORKS

Internal code number of network	Concentration of guanidinoacetic acid (GAA)			
	Baseline	1 µM	10 µM	100 µM
24250	81,31	64,48	22,95	0
20554	96,66	103,94	45,98	20,92
20512	235,68	146,4	108,13	42,67
18333	112,49	107,47	38,17	79,71
18332	162,97	138,66	126,47	135,5
18331	236,94	254,15	169,2	140,85
15345	136,69	82,65	62,04	0
