# Supplementary material for: Lack of Epileptogenic Effects of the Creatine Precursor Guanidinoacetic Acid on Neuronal Cultures In Vitro
Source: Biomolecules. 2022 Dec 30;13(1):74. doi: 10.3390/biom13010074 (PMC9856136; doi:10.3390/biom13010074)
Supplement: Supplementary file 1 [file biomolecules-13-00074-s001.zip › Supplemental table 4.pdf]

SUPPLEMENTAL TABLE 4: Mean frequency intra burst (MFIB) of single networks - Measurement unit: spikes/mSec

NEOCORTICAL NETWORKS

Internal code number of network	Concentration of guanidinoacetic acid (GAA)			
	Baseline	1 µM	10 µM	100 µM
15345	0,15	0,15	0,17	0,04
18331	0,19	0,21	0,22	0,21
18332	0,19	0,2	0,21	0,19
19216	0,13	0,13	0,11	0,09
20551	0,16	0,12	0,14	0,08
20559	0,12	0,11	0,11	0
22643	0,1	0,01	0,01	0
20554	0,16	0,17	0,14	0

HIPPOCAMPAL NETWORKS

Internal code number of network	Concentration of guanidinoacetic acid (GAA)			
	Baseline	1 µM	10 µM	100 µM
24250	0,11	0,1	0,03	0
20554	0,12	0,11	0,07	0,01
20512	0,1	0,13	0,14	0,11
18333	0,13	0,15	0,04	0,08
18332	0,08	0,09	0,1	0,09
18331	0,1	0,1	0,12	0,12
15345	0,12	0,11	0,07	0
