# Supplementary material for: Lack of Epileptogenic Effects of the Creatine Precursor Guanidinoacetic Acid on Neuronal Cultures In Vitro
Source: Biomolecules. 2022 Dec 30;13(1):74. doi: 10.3390/biom13010074 (PMC9856136; doi:10.3390/biom13010074)
Supplement: Supplementary file 1 [file biomolecules-13-00074-s001.zip › Supplemental table 6.pdf]

SUPPLEMENTAL TABLE 6 - EFFECTS OF 10µM GUANIDINOACETIC ACID (GAA) ON NUMBER AND LATENCY OF STIMULUS-EVOKED SPIKES (PAIRED DATA)

Number of stimulus-evoked spikes within 400 mSec from electrical stimulus

Pre GAA	Post GAA
1,55	1,54
2,6	2,3
2,63	2,45
1,89	1,89
3,76	2,65

Latency of stimulus-evoked spike (milliseconds)

Pre GAA	Post GAA
276,6	257,46
197,61	245,79
227,07	226,11
279,66	334,35
223,82	157,14
