# Supplementary material for: Lack of Epileptogenic Effects of the Creatine Precursor Guanidinoacetic Acid on Neuronal Cultures In Vitro
Source: Biomolecules. 2022 Dec 30;13(1):74. doi: 10.3390/biom13010074 (PMC9856136; doi:10.3390/biom13010074)
Supplement: Supplementary file 1 [file Supplemental table 5.pdf › Supplemental table 5.pdf]

SUPPLEMENTAL TABLE 5 - EFFECTS OF DAYS IN VITRO AND OF GUANIDINOACETIC ACID ON VARIOUS ELECTROPHYSIOLOGICAL PARAMETERS

Controls - no guanidinoacetic acid added

Internal code of network - DIV	EL	MFR (sp/s)	MBR (burst /min)	BD (ms)	MFIB (spike/ ms)	DIV	EL	MFR (sp/s)	MBR (burst /min)	BD (ms)	MFIB (spike /ms)	DIV	EL	MFR (sp/s)	MBR (burst/ min)	BD (ms)	MFIB (spike/ ms)
**14986 - DIV 11**	46	2,46	5,12	333,27	0,04	**DIV 18**	57	8,56	11,85	293,01	0,14						
**18882 - DIV 11**	26	2,96	6,28	298,37	0,06												
**34340 - DIV 11**	40	4,21	11,84	317,15	0,04	**DIV 18**	55	6,24	14,51	184,7	0,11						
**34341 - DIV 11**	46	2,3	5,64	289,64	0,04	**DIV 18**	57	4,84	10,95	195,7	0,1	**DIV 25**	55	6,99	12,1	250,99	0,13
**38925 - DIV 11**	48	3,36	11,6	301,57	0,04	**DIV 18**	58	4,95	4,59	499,39	0,13	**DIV 25**	24	0,61	1,11	228,99	0,08

Guanidinoacetic acid (GAA) 10uM

Internal code of network - DIV	EL	MFR (sp/s)	MBR (burst /min)	BD (ms)	MFIB (spike/ ms)	DIV	EL	MFR (sp/s)	MBR (burst /min)	BD (ms)	MFIB (spike /ms)	DIV	EL	MFR (sp/s)	MBR (burst/ min)	BD (ms)	
**7094 - DIV 11**	13	2,97	2,41	220,04	0,03	**DIV 18**	45	3,62	4,12	596,83	0,08	**DIV 25**	46	5,34	5,5	437,93	0,1
**34347 - DIV 11**	46	2,53	4,93	261,75	0,04	**DIV 18**	57	4,03	12,42	195,05	0,08						
**38927 - DIV 11**	13	6,7	8,63	345,75	0,09												
**38934 - DIV 11**	9	2,21	4,14	224,06	0,03												

Abbreviations: DIV=Days in vitro; EL: Number of active electrodes in the array; MFR=Mean firing rate; MBR=Mean bursting rate; BD=Burst duration; MFIB=Mean firing intra burst; sp=spikes; s=seconds; min=minute; ms=millisecond
